# Novel definition of time range and risk factors of pregnant women with gestational diabetes mellitus detected early in pregnancy a cluster analysis using clinical data of the German GestDiab cohort

**DOI:** 10.1186/s13098-025-02000-3

**Published:** 2025-11-14

**Authors:** Isabel Sontag, Maik Kschischo, Matthias Kaltheuner, Luise Jander, Philipp Leubner, Heinke Adamczewski, Dietmar Weber, Annette Hasenburg, Henning E. Adamek, GestDiab Group, M. Behling, R. Betzholz, M. Gierse, J. Klein, S. Mohan, D. Weber

**Affiliations:** 1 Department of Endocrinology and Diabetology, Med. Klinik 2, Klinikum Leverkusen, Academic Hospital of the University of Cologne, 51375 Leverkusen, Germany; 2 Department of Computer Science, University of Koblenz, Koblenz, Germany; 3 Department of Mathematics, Informatics and Technology, University of Applied Sciences Koblenz, Remagen, Germany; 4 winDiab gGmbH, Scientific Institute of Resident Diabetologists, Neuss, Germany; 5 University Medical Center of the Johannes Gutenberg University Mainz, Mainz, Germany

**Keywords:** Cluster analysis, Data-driven Clustering, Early gestational diabetes mellitus, Gestational diabetes mellitus, Machine learning

## Abstract

**Background:**

Gestational diabetes mellitus (GDM) is the most common pregnancy complication worldwide, leading to a variety of short and longterm complications for both mother and child. International screening and diagnostic recommendations remain disputed and incoherent. A high proportion of women with GDM can be detected early in pregnancy. However, there is no consensus about diagnosis of GDM in early pregnancy. In this study, we aimed to detect a clear time frame for early GDM (eGDM). Based on these results, we compared the characteristics of early vs standard GDM.

**Methods:**

In this secondary data analysis all data were sourced from diabetes specialist practices, from Germany and were collected between the years of 2018–2021.We applied k-means clustering to create two homogenous groups, identifying an early and a standard GDM cohort. Subsequently, we analyzed presented data regarding its association with early gestational diabetes (eGDM) and standard GDM (sGDM).Finally, a prediction model was developed using a set of nine variables. Odds ratios of each variable served as an independent indicator on the individual effect of each factor.

**Results:**

Our dataset included 18,495 pregnancies complicated by gestational diabetes. The decision boundary through our k-means analysis was determined as 20.88 week of gestation. Both groups had a mean age of 33 years of age. Women with early gestational diabetes presented higher pre-pregnancy body weight (86.6 kg vs. 76.8 kg) and higher pre-pregnancy BMI (31.1 vs. 27.9 kg/m^2^) and with an average weight difference of 9.8 kg. Fasting plasma glucose differed significantly between both groups (eGDM: 98.1 mg/dl [5,4 mmol/l] vs. sGDM 94.7 mg/dl [5,3 mmol/l]). The logistic regression model for eGDM achieved an area under the curve of 0.83.

**Conclusions:**

We defined early gestational diabetes as gestational diabetes occurring before 21st week of gestation. Fasting plasma glucose with a threshold value of 98 mg/dl [5,4 mmol/l] could be an appropriate tool for screening.

**Trial registration:**

GestDiab is listed in the German Trial Registry (https://registersuche.bqs.de/search.php)

**Supplementary Information:**

The online version contains supplementary material available at 10.1186/s13098-025-02000-3.

## Introduction

Gestational diabetes is defined as hyperglycemia detected during pregnancy that does not meet the diagnostic criteria for overt diabetes mellitus in pregnancy [1] It is the most common medical complication in pregnancy, with a staggering incidence between 9.3%—25% of all pregnancies worldwide [2, 3] In 2016 approximately 15.1% of all pregnancies in Germany were complicated by GDM [4] Typically, the maternal hyperglycemic state resolves after birth*.*[5] Women affected by GDM have an estimated ten-fold increase of risk of developing Diabetes mellitus Type 2 (T2D) later in life highlighting its tight association with other metabolic diseases [6]. 

A variety of risk factors predisposing to GDM have already been identified, which include: GDM in a previous pregnancy, advanced maternal age, family history of T2D, obesity and ethnicity [7].

GDM leads to a variety of maternal and neonatal cardio-metabolic complications [8] The landmark „Hyperglycemia and Adverse Pregnancy Outcome (HAPO)“-study found an association between maternal hyperglycemia and increase of pre-eclampsia, preterm delivery, cesarean section, large for gestational age (LGA) infants, shoulder dystocia, neonatal hypoglycemia, hyperbilirubinemia and neonatal intensive care unit (NICU) admission [9].

Based on recommendations of international associations the diagnosis can be confirmed between week 24–28 of gestation by an oral glucose tolerance test (OGTT) and alternatively by HbA1c or fasting plasma glucose (FPG) [8] Women with risk factors are advised to test for GDM prior to the recommended time range of 24th-28th week of gestation [10, 11].

In light of earlier screening, a new subset of GDM has emerged over the years: Hyperglycemia in early pregnancy. In literature, eGDM is being referred to as GDM diagnosed before the 24th week of gestation [7] Although based on weak evidence, it has recently been often referred to as GDM before the 20th week of gestation [12] The eGDM subgroup appears to be more similar to the T2DM rather than the sGDM cohort diagnosed after 24th week of gestation. The eGDM subgroup therefore emerges to be the key risk group in its own right within hyperglycemic disorders [13].

The lack of established international diagnostic criteria for eGDM continues to complicate matters further [14] Current IADPSG Guidelines with an FPG diagnostic threshold of 92 mg/dl [5.1 mmol/l] do not appear to be useful for early testing as a result of progressively decreasing blood glucose values during early gestation [15].

The TOBOGM-study has become a landmark within the field of eGDM-research. This international randomized controlled trial (RCT) divided pregnant women with diagnosed eGDM into two groups: one group received immediate treatment, the control group received retesting during 24–28 weeks of gestation, and treatment was initiated if sGDM was diagnosed. This study demonstrated improved composite neonatal outcomes (birth before 37 weeks gestation, birth weight of > 4500 g, birth trauma, neonatal respiratory distress, phototherapy, stillbirth or neonatal death and shoulder dystocia) when eGDM was diagnosed and treated immediately [16].

Currently, there is no consensus about diagnosis of GDM in early pregnancy. In this study, we aimed to detect a clear time frame for early GDM (eGDM). Based on these results, we compared the characteristics of early vs standard GDM.

## Methods

### Study population

The ‘GestDiab’ register was launched in 2004 as a project coordinated by the scientific institute of office-based diabetologists that monitors the treatment of pregnant women with hyperglycemia in diabetes specialist practices (DSPs) [17].

According to the Clinical Practice Guidelines of the German Diabetes Association screening for risk of diabetes at first medical appointment in pregnancy is advised for the following risk factors: Pregnancies with previous GDM, age > 35 years. 1 st and 2nd degree relatives with diabetes, ovulation induction, origin in Asian region, BMI > 30 [18] Patients were tested with a three-point 75 g oral glucose tolerance test (OGTT). GDM is confirmed if any of the following venous plasma glucose values are met or exceeded: fasting: 92 mg/dl [5.1 mmol/L], 1 h: 180 mg/dl [10.0 mmol/L], and 2 h: 153 mg/dl [8.5 mmol/L}.

The GestDiab-register was approved by the ethics committee of the Medical Association of North Rhine (Ethics Committee No.: 2019272). The use of register data is in line with the common data protection regulations system [19].

All participating pregnant women received written information on the project and gave written consent to enter their data and the data of their newborn in pseudonymized form into the GestDiab database. The study was performed in accordance to the Declaration of Helsinki.

A dataset of 26,025 patients was at our disposal which contained all pregnancies with hyperglycemia diagnosis given between 2018 and 2021. The following predictor variables were included: pre-pregnancy body weight, BMI, previous history of GDM, family history of first degree relatives regarding diabetes, glucose levels at OGTT, baseline HbA1c, maternal age, parity and gravidity. Due to the nature of the present study, detailed information about ethnicity and socioeconomic status of the included patients was not available. We excluded participants with diagnosed prevalent Diabetes mellitus (except for GDM) of any type, women with overt diabetes in pregnancy, and those with a missing value for any of the exposures, outcomes or potential confounders. This resulted in the exclusion of in total 7530 women. In total we processed a dataset of 18,495 cases.

### Clustering

Clustering techniques are widely used in data analysis and machine learning to group a set of objects in such a way that objects within the same group (or cluster) are more similar to each other than to those in other groups. The goal is to partition data into meaningful or useful clusters based on their inherent patterns or similarities [20] K-means is a commonly used technique which partitions all objects in a given data set into K distinct groups (clusters) and represents each cluster by a centroid, which can be viewed as a type of prototype for the corresponding cluster.

Here, we used k-means clustering to group all women with GDM diagnosis into two (k = 2) distinct groups characterized by an early GDM diagnosis and a standard GDM diagnosis. The only variable used for clustering was the time of diagnosis.

### Statistical analysis

Data has been presented as means, with a 95% confidence interval for continuous variables, and as both frequences and percentages for categorical variables. We used a contingency table method with Fisher’s test for categorical variables. A two-tailed p-value of 0.05 was considered statistically significant.

The p-value generally depends on both the effect size and the variability of the estimated variables, for example means or differences of means. The variability of the estimates decreases with increasing sample size and the p-values also typically decrease. This renders many statistical tests statistically significant, even if the actual effect size is small and possibly not relevant from a medical point of view. To address this issue we computed effect size estimates [21] Cramer’s V was used to quantify the association between categorical variables. Here, 0 indicates a weak association and 1 a strong association. For pairwise comparisons of numerical variables in two different groups we used the Wilcoxon rank-sum test for paired samples and quantified the effect sizes by the rank-correlation coefficient r (small effect: 0 < r < 0.3; medium effect: 0.3 < r < 0.5; large effect: r > 0.5). Data has been plotted on a logarithmic scale (common logarithm).

A Logistic Regression model was created for classifying eGDM versus sGDM. To select the most relevant predictor variables, we performed forward–backward selection and cross validation on a training set consisting of 80% of the data. To assess the performance of the logistic regression classifier we used an independent testing set of 20% of the data. Receiver operating characteristic (ROC) curves and the respective area under the curve was used as a measure of predictive performance. The odds ratios (OR) of each predictor variable provided information about the individual effects of each factor along with helping to identify key risk factors.

## Results

### Cluster analysis

**Fig. 1:**
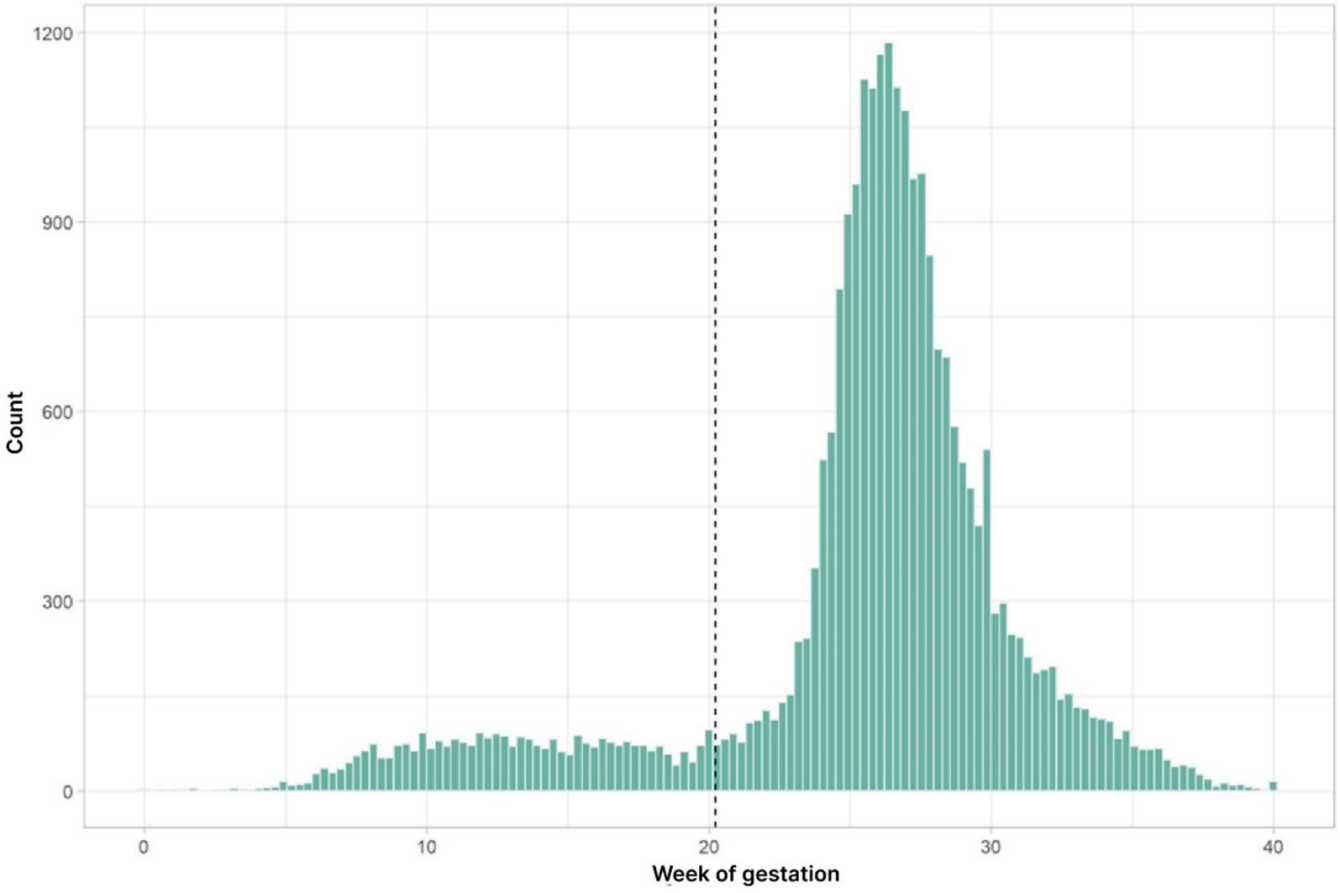
A histogram for the absolute frequency of GDM diagnosis as a function of pregnancy weeks. The women were grouped into two distinct sub-groups with an early and a late diagnosis using k-means clustering. The dashed line indicates this partition with the early/standard diagnosis group defined by a GDM diagnosis before/after week 20.88

The frequency of GDM-diagnosis exhibits two distinct peaks of one around 12 and another of 27 weeks of pregnancy (Fig. 1). This observation allowed us to group all women with GDM in our data set into two distinct groups of either an eGDM or a sGDM. We used k-means clustering (k = 2 clusters) for the time of diagnosis to define a decision boundary, which was found to be approximately week 20.88 of pregnancy. Based on these observations we assigned all women with a GDM before week 20.88 as early GDM cases and all women diagnosed after this time point were considered as standard GDM. Altogether, we classified 1,639 patients as eGDM and 16,856 patients as sGDM.

### Risk factors

We then compared maternal characteristics between the two clusters. These were balanced according to the discrimination of the cluster analysis (Table 1). Women with eGDM were more likely to present with a higher pre-pregnancy body weight (86.65 kg vs. 76.81 kg) and higher pre-pregnancy BMI (31.15 vs. 27.99 kg/m^2^). Weight appears to be one of the major risk factors according to our data, the eGDM group was 9.841 kg heavier prior to gestation compared to the sGDM counterpart. Maternal weight at the time of eGDM diagnosis however served no predictive value in our study.

**Table 1:** Maternal characteristics and laboratory results, by GDM type

VARIABLE	eGDM		sGDM		
	Mean value	95% CI	Mean value	95% CI	p-values
week of gestation at time of diagnosis	13.936	[13.752;14.119]	27.231	[27.187;27.276]	< 0.001
HbA1c (%)	5.222	[5.205;5.239]	5.194	[5.189;5.2]	0.0019
Height (cm)	166.553	[166.224;166.881]	165.485	[165.384;165.585]	< 0.001
Pre-pregnancy weight (kg)	86.654	[85.551;87.757]	76.813	[76.528;77.098]	< 0.001
Weight at time of diagnosis (kg)	88.784	[87.699;89.869]	85.024	[84.747;85.301]	< 0.001
BMI kg/m^2^	31.153	[30.787;31.519]	27.994	[27.897;28.09]	< 0.001
Gravidity	2.685	[2.613;2.756]	2.273	[2.25;2.295]	< 0.001
Parity	1.270	[1.217;1.323]	0.970	[0.952;0.988]	< 0.001
Age	33.231	[32.999;33.462]	32.630	[32.552;32.707]	< 0.001
FPG mg/dl	98.067	[97.613;98.522]	94.663	[94.518;94.808]	< 0.001
oGTT 1h-blood glucose value mg/dl	158.270	[156.428;160.113]	171.243	[170.756;171.73]	< 0.001
oGTT 2h-blood glucose value mg/dl	124.284	[122.84;125.727]	133.982	[133.565;134.399]	< 0.001
Random blood glucose mg/dl	104.726	[102.352;107.1]	110.942	[110.106;111.778]	< 0.001

For all variables, a one-sample t-test was applied separately to the eGDM and sGDM groups, but only for the purpose of calculating confidence intervals. We also present p-values to compare the means of two groups

The higher the body weight of the participants, the more likely the occurrence of eGDM (Cramers V = 0.136). The occurrence of eGDM increased almost linearly with the weight class of the pregnant women. The highes occurance of eGDM was observed in the weight class of obesity class 3 ((BMI > 40 kg/m^2^) n = 1093; eGDM 19.95%).

The fasting OGTT value was significantly higher in the eGDM cohort (eGDM: 98.064 mg/dl vs. GDM 94.663 mg/dl). Within the ranking of numerical variables, fasting glucose emerged as one of the strongest predictive values for eGDM manifestation. According to the Wilcoxon rank sum test, the effect size is 0.102, indicating a mild effect. The other OGTT values (1-h and 2-h values) showed a contrasting pattern and were consistently higher in participants with sGDM rather than eGDM. An eGDM diagnosis during pregnancy was also associated with a higher HbA1c of 5.22%. In comparison, individuals with sGDM had an average HbA1c of 5.194%. Among other variables, HbA1c appears to be a relatively weaker predictor for eGDM.

Maternal age differed insignificantly; women with eGDM were 0.601 years older compared to those with sGDM. Both groups had a mean age of 33 years. However, the difference is still too small to serve as predicative factor (very low effect size of 0.03).

Gravidity and parity were significantly higher in the eGDM cohort compared to the sGDM cohort. For 6284 Patients, it was their first pregnancy whilst a history of previous pregnancies was more common in the eGDM group (2.685) compared to the sGDM group (2.273). Therefore, parity was also increased in the eGDM cohort (eGDM: 1.270 vs. GDM 0.970).

One of the strongest associations with eGDM proved to be the previous history of GDM (Cramers V = 0.330). Of the 2.887 women diagnosed with a previous history of GDM, 831 developed eGDM (28.78%) compared to women without a previous history (4.67%). It was also the strongest predictive factor amongst the categorial variables.

Of the 5930 study participants with a positive family history of diabetes mellitus, 10.57% (n = 625) developed eGDM. Among 10,933 patients without a family history, 7.85% (n = 858) developed eGDM. According to the Cramers V test, the association to eGDM was 0.045, indicating no statistical association.

### Prediction model for early GDM and Odds ratios

A binary logistic regression model for classifying pregnancies into eGDM versus sGDM was developed. A variable selection method identified nine predictor variables that, together, can predict early or standard GDM. These variables were included in the final logistic regression model, namely: BMI, maternal age, GDM in previous pregnancy, family history of diabetes mellitus, FPG, 1 h -and 2 h plasma glucose values, gravidity and parity (Table 2).

**Table 2:** Odds plot parameters making up the odds plot

Variables	Odds ratio	Lower CI	Upper CI
Gravidity	1.0529951	0.9823632	1.1274793
BMI kg/m^2^	1.0425321	1.0318767	1.0532481
Age	1.0239866	1.0088398	1.0394169
FPG mg/dl	1.0228518	1.0148958	1.0308846
oGTT 1h-blood glucose mg/dl	0.9880441	0.9857204	0.9903579
oGTT 2h-blood glucose mg/dl	0.9947157	0.9918974	0.9975352
Family history of T2D	0.9115647	0.7898771	1.0528991
Parity	0.8858021	0.8006705	0.9792161
Previous GDM	0.1134984	0.1134984	0.1317620

**Fig. 2:**
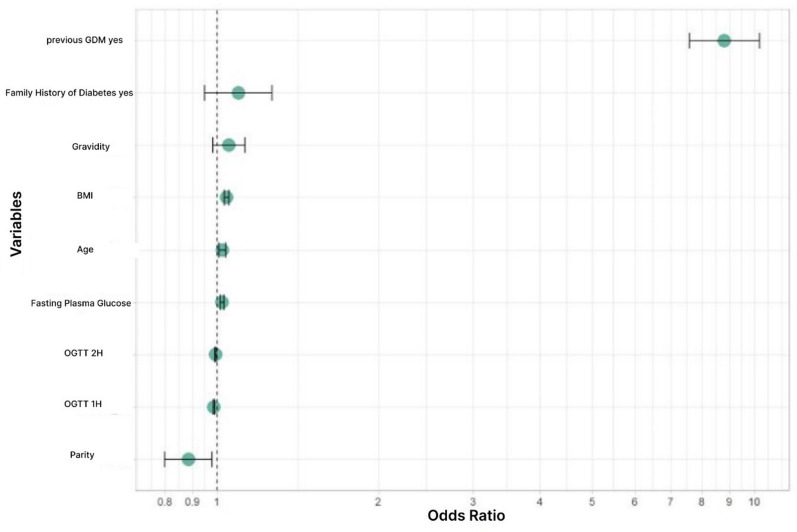
Odds Plot. Influence of increasing each variable by one Unit and therefore increasing the likelyhood of developing eGDM

We used odds ratios to quantify the predictive importance of each single variable (Fig. 2). For numerical variables, the odds ratio represents the change in risk for early versus standard GDM with a one-unit increase in the variable, when the other variables are kept constant. For binary variables, it shows the change in risk when the feature is present versus absent, when again all other variables are kept constant. Please note, that the odds ratios cannot directly be compared with each other, because a unit change in one variable has a different physical unit than a unit change in another variable.

GDM in a previous pregnancy (OR: 0.113; 95% CI 0.113–0.131) is significantly associated with a risk of developing eGDM. BMI (OR: 1.042; 95% CI 1.031–1.053) gravidity (OR: 1.052; 95% CI 0.982—1.127), FPG (OR: 1.022; 95% CI 1.014–1.030) and maternal age (OR 1.023; 95% CI 1.030–1.0394) are also associated with an increased risk of eGDM.

The odds ratios for a family history of diabetes mellitus (OR: 0.911; 95% CI 0.789—1.052), gravidity (OR: 1.052; 95% CI 0.982–1,127), parity (OR: 0.885), 1 h-plasma glucose level (OR: 0.988) and 2 h-plasma glucose level (OR: 0.994) were not significantly different from one at the 5% level in this multivariate logistic regression model. This indicates that these variables exhibit a high degree of variablity and are therefore not robust enough as markers for eGDM. However, when these variables were removed from the logistic regression model, the validation set accuracy of the prediction model decreases. This indicates that they still have some predictive value in addition to the variables with significant odds ratios. Therefore, we included them in the multivariate logistic regression model.

**Fig. 3:**
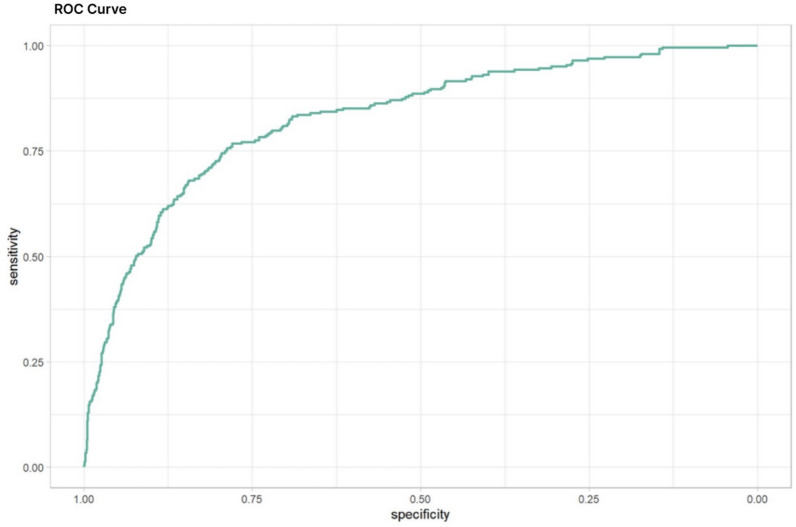
ROC Curve. The ROC curve for the logistic regression model, incorporating variables like BMI, age, prior GDM, family history of diabetes, fasting OGTT, 1-h and 2-h OGTT values, gravidity, and parity, achieved an AUC of 0.8298 (95% CI: 0.8027–0.857, Sensitivity: 0.7680608, Specificity: 0.779) indicating a moderately good predictive value

The classification performance of the resulting logistic was evaluated by a ROC curve indicating the tradeoff between sensitivity and specificity (Fig. 3). The area under the curve (AUC) of 0.83 (95% CI: 0.8027–0.857, sensitivity: 0.7680608, Ssecificity: 0.779) indicates a moderately good classification performance.

In summary, women with eGDM had higher pre-pregnancy body weight and BMI. They also had higher gravidity, parity, and a history of GDM in a previous pregnancy. The definition of eGDM as any GDM diagnosed before the 21 st week of gestation has now been validated by a robust dataset for the first time. A fasting plasma glucose level of 98 mg/dl [5,4 mmol/L] could serve as an effective screening tool for eGDM.

## Conclusions

Gestational Diabetes continues to be an increasing health burden worldwide. By week 15 of gestation, increased insulin values can already be detected in amniotic fluid of GDM-Pregnancies [22] Later on, increased fetal growth as a result of maternal hyperglycemia can be observed [23] Additionally, the results of the HAPO-Follow up study (HAPO-FUS) suggest hyperglycemia exerting an even greater influence on the long-term metabolic health of children than previously understood.

International guidelines continue to overlook the growing evidence of the heterogenous nature of GDM and hold on to an outdated screening schedule [24].

The first summit discussing eGDM in 2022 largely agreed on testing and treating eGDM due to the evident risks of early hyperglycemia. However, no diagnostic criteria for early testing has been defined, citing again the lack of scientific verification. The definition of eGDM has been set to GDM ≤ 20 week of gestation. Additionally, 75 h oGTT (oral glucose tolerance test) has been found to be the only option for early testing due to lacking studies regarding other diagnostic tests [25].

Data driven AI will most likely revolutionize healthcare in upcoming years in various aspects of medicine. For example, facilitating the monitoring of patients (ie. by wearables), using algorithms to proactively identify pathologies and alter therapeutic interventions towards more personalised medicine. [26, 27].

In terms of (e)GDM data driven AI has already been used to effectively predict maternal risk, filter out risk factors and by monitoring blood glucose via continuous glucose monitoring (CGM) [28].

Recently, Salvatori et al. used data-driven cluster analysis to identify novel subgroups of GDM. They detected three different clusters, each characterised by distinct BMI-values and different tendencies towards glucose lowering drugs being applied during pregnancy [29] Aforementioned studies highlight the growing novel possibilities arising with digitalisation around the globe.

We aimed to integrate a dataset of 18,495 pregnancies into the current body of evidence. By using k-means artificial intelligence we sought to validate the current trend to further define eGDM. Based on two homogenous clusters we can now confidently define eGDM as GDM before the 21 st week of gestation. To our knowledge, this is the first time a large dataset confirms the currently proposed definition of early GDM. As a matter of fact, the findings appear to have clinical plausibility. Our findings build on the previous study done by Simmons et al. [16] in which GDM diagnosis was performed at 20 weeks of gestation, following WHO recommendations, and provides a clear definition of eGDM based on extensive data analysis.

Our second goal was to stratify the risk factors specific to the complication-prone eGDM.

A key distinguishing variable in our study was the considerably elevated FPG in the eGDM (eGDM: 98.067 mg/dl (95% KI 97.613; 98.522) [5,4 mmol/l) vs. sGDM: 94.663 mg/dl (94.518;94.808)[5,3 mmol/l) cohort at time of diagnosis, meaning the fasting OGTT value was nearly 6 mg/dl higher than the IADPSG recommended fasting OGTT value of 92 mg/dl [6,1 mmol/l]. The 1 h and 2 h plasma glucose values however were significantly lower than the IADPSG thresholds for the 24th to 28th week of gestation. Our OGTT data from 18,495 participants suggest that an eGDM diagnosis requires its own reference values, tailored to the blood glucose dynamics during pregnancy. An FPG is readily available and inexpensive. Powered by the right diagnostic thresholds FPG could be an appropriate screening test with a cut-off value of 98 mg/dl [5,4 mmol/l]. A pragmatic approach until more research is available, would be to treat pregnant women in only higher pathological FPG values, and retest those with lower-pathological values around week 24.−28. of gestation [16] A recommendation for action in case of FPG levels beteen 92 [5,1 mmol/l] and 124 mg/dl [6,9 mmol/l] is currently under discussion. According to our study, the HbA1c is not appropriate for early testing [30] On a pathophysiological level, this seems to be validated by the heightened erythrocyte turnover in early pregnancy, thereby decreasing this marker during this period of time [31] Additionally, the need for different cut-offs for different ethnicities further complicates its use as a universal screening tool*.*[32] Global screening strategies with sensitive biomarkers must currently be ruled out primarily in terms of cost [33].

Pre-pregnancy overweight women are at an increased risk of developing early onset gestational diabetes. GDM likely occurs far earlier than the 24th week of gestation due to pre-existing insulin resistance associated with obesity. Additionally, a history of a previous pregnancy complicated by GDM significantly predisposes women to eGDM [34] They also presented with increased gravidity and parity, in line with previous literature [13] Interestingly, a family history of diabetes mellitus revealed only limited predictive features in our study.

Finally, we developed a prediction model based on nine variables from our study. The relatively simple collection allows for the creation of a robust ROC curve, with an area under the curve (AUC) amounting to 0.829 (95% CI: 0.8027–0.857; sensitivity 0,768; specifity 0.779), which is considered to be a moderately good value for eGDM prediction. This data can be gathered during routine clinical visits in early pregnancy, determining the need for earlier blood glucose monitoring. Similar prediction models have included rather difficult to acquire variables, for example insulin values were used in a prediction model from Wu et al., with an AUC of 0.872 [35] Other AUCs in literature had reported weaker predictive values than ours despite readily available variables, for example Guo et al. showcased an AUC of 0.7 [36].

A limitation, however, is that the original purpose and research questions of the data were not specifically designed for subsequent analyses[37] This was a register study, and the reason why patients were included is not determined. However, the clear distinction with the k-means clustering analysis suggests that eGDM and sGDM can be discriminated at about 20 weeks.

Our findings recommend the definition of eGDM as any GDM diagnosed before the 21 st week of gestation; this insight has now been validated by a robust dataset for the first time. A fasting plasma glucose level of 98 mg/dl [5,4 mmol/L] could serve as an effective screening tool for eGDM.

Early detection of GDM is a shared challenge that requires a scientifically sound strategy to target risk groups and circumstances irrespective of cultural and socio-economic differences.

## Supplementary Information

Additional file 1.

Additional file 2.

## Data Availability

The datasets generated and analysed during the current study are not available publicly as they are subject to national data protection laws and restrictions imposed by the ethics committee to protect the privacy of study participants.
