# Supplementary material for: Novel definition of time range and risk factors of pregnant women with gestational diabetes mellitus detected early in pregnancy a cluster analysis using clinical data of the German GestDiab cohort
Source: Diabetol Metab Syndr. 2025 Nov 14;17:426. doi: 10.1186/s13098-025-02000-3 (PMC12616929; doi:10.1186/s13098-025-02000-3)
Supplement: Supplementary file 1 — Additional file 1. [file 13098_2025_2000_MOESM1_ESM.docx]

Ethical Approval/Exemption Document

The GestDiab-register was approved by the ethics committee of the Medical Association of North Rhine (Ethics Committee No.: 2019272). The use of register data is in line with the common data protection regulations system.^[[1]](#endnote-1)^
All participating pregnant women received written information on the project and gave written consent to enter their data and the data of their newborn in pseudonymized form into the GestDiab database. The DSPs took part on a purely voluntary basis. The study was performed in accordance to the Declaration of Helsinki.

1. Linnenkamp U, Greiner GG, Haastert B, et al. Postpartum screening of women with GDM in specialised practices: Data from 12,991 women in the GestDiab register. *Diabet Med*. 2022;39(7):e14861. doi:10.1111/dme.14861 [↑](#endnote-ref-1)
