# Supplementary material for: Novel definition of time range and risk factors of pregnant women with gestational diabetes mellitus detected early in pregnancy a cluster analysis using clinical data of the German GestDiab cohort
Source: Diabetol Metab Syndr. 2025 Nov 14;17:426. doi: 10.1186/s13098-025-02000-3 (PMC12616929; doi:10.1186/s13098-025-02000-3)
Supplement: Supplementary file 2 — Additional file 2. [file 13098_2025_2000_MOESM2_ESM.pdf]

Ärztekammer Nordrhein
Körperschaft des öffentlichen Rechts

Ärztekammer Nordrhein, Tersteegenstraße 9, 40474 Düsseldorf

Herrn
Dr. iur. Tobias Jacquemain, LL.M.
Datenschutzbeauftragter der
winDiab gGmbH
Severinstr. 53
50678 Köln

ETHIK-KOMMISSION

Ansprechpartner/in:
Sandra Franz
ethik@aekno.de
Tel 0211 4302 – 2286
Fax 0211 4302 – 2279

Ihr Schreiben vom:	Ihr Zeichen:	Unsere lfd. Nummer:	Datum:
01.11.2019	-	2019272	07.11.2019

Berufsrechtliche Beratung nach § 15 Berufsordnung vor der Durchführung biomedizinischer Forschung am Menschen

GestDiab Register
Diabetes in der Schwangerschaft

Sehr geehrter Herr Dr. Jacquemain,

die Ethik-Kommission der Ärztekammer Nordrhein hat Ihr oben genanntes Schreiben vom 01.11.2019 mit der Patienteninformation und Einverständniserklärung vom 01.11.2019 und dem Registerprotokoll Version 1.1 vom 09.10.2019 zur Kenntnis genommen.

Damit konnten Sie alle Punkte unseres Votums vom 02.09.2019 zufriedenstellend umsetzen, so dass keine berufsethischen und berufsrechtlichen Bedenken mehr gegen die Durchführung der Studie bestehen.

Vorsorglich weist die Ethik-Kommission darauf hin, dass die ärztliche und juristische Verantwortung für die Durchführung der Studie uneingeschränkt bei Ihnen und Ihren Mitarbeitern verbleibt.

Wir machen darauf aufmerksam, dass sich das oben genannte Votum nur auf die bisher eingereichten Unterlagen bezieht. Nachträgliche Änderungen in Organisation und Ablauf der klinischen Prüfung, die nach Ihrer Auffassung vom Votum der Ethik-Kommission umfasst sein sollen, sollten umgehend zur Beratung mitgeteilt und die geänderten Passagen deutlich kenntlich gemacht werden.

Informationen und Änderungen, zu denen Sie kein Votum der Ethik-Kommission mit einem entsprechenden Antrag erbitten möchten, sind demgegenüber nicht zuzusenden.

Tersteegenstraße 9
40474 Düsseldorf

Postfach 30 01 42
40401 Düsseldorf

Telefon 0211 4302-0
Fax 0211 4302-2009
Mail aerztekammer@aekno.de
Web www.aekno.de

Kernarbeitszeiten:
Mo. bis Do. 9 Uhr bis 15 Uhr
Freitag 9 Uhr bis 14 Uhr

Bankverbindung:
Deutsche Apotheker- und Ärztebank eG, Düsseldorf
IBAN DE89 3006 0601 0001 1452 90
BIC DAAEDEDDXXX

TÜVRheinland CERT ISO 9001

Wir gehen davon aus, dass Sie die Prüfer in unserem Kammerbezirk über dieses Votum informieren.

Mit freundlichen Grüßen
gez.

Prof. Dr. med. K. Racké
Vorsitzender des Gremiums I
der Ethik-Kommission

Ausgefertigt:
i. A.

Julia Rümler
Rechtsreferentin der Ethik-Kommission

Medical Association of North Rhine, Tersteegenstraße 9, 40474 Düsseldorf

To
Dr. iur. Tobias Jacquemain, LL.M.
Data Protection Officer of
winDiab gGmbH
Severinstr. 53
50678 Cologne

ETHICS COMMITTEE
Contact Person:
Sandra Franz
ethik@aekno.de
Tel 0211 4302 - 2286
Fax 0211 4302 - 2279

Your Letter of: 01.11.2019
Your Reference: -
Our Reference: 2019272

Professional Ethical Consultation according to § 15 Professional Code prior to the
Conduct of Biomedical Research on Humans
GestDiab Register
Diabetes in Pregnancy

Date: 07.11.2019

Dear Dr. Jacquemain,

The Ethics Committee of the Medical Association of North Rhine has acknowledged your above-mentioned letter dated 01.11.2019, along with the patient information and consent form dated 01.11.2019 and the register protocol Version 1.1 dated 09.10.2019.

You have satisfactorily addressed all the points of our opinion dated 02.09.2019, so that there are no longer any professional ethical and legal objections to conducting the study.

As a precaution, the Ethics Committee points out that the medical and legal responsibility for conducting the study remains fully with you and your staff.

We would like to point out that the aforementioned opinion only applies to the documents submitted so far. Any subsequent changes in the organization and procedure of the clinical trial, which in your view should be covered by the Ethics Committee's opinion, should be communicated immediately for consultation, and the altered sections should be clearly marked.

Information and changes for which you do not seek an opinion from the Ethics Committee with a corresponding application, on the other hand, do not need to be submitted.

We assume that you will inform the investigators in our chamber district about this vote.

Sincerely,

Signed: Issued:

Prof. Dr. med. K. Racké
Chairman of Committee I of the Ethics Committee

Julia Rümler
Legal Advisor
